# Molybdenum-Oxide-Modified PEDOT:PSS as Efficient Hole Transport Layer in Perovskite Solar Cells

**DOI:** 10.3390/molecules29215064

**Published:** 2024-10-26

**Authors:** Pu Fan, Zhipeng Zhou, Jianghao Tian, Junsheng Yu

**Affiliations:** 1 Yangtze Delta Region Institute Huzhou, University of Electronic Science and Technology of China, Huzhou 313001, China; fanpu@csj.uestc.edu.cn (P.F.); 202322050612@std.uestc.edu.cn (Z.Z.); 202352050625@std.uestc.edu.cn (J.T.); 2 State Key Laboratory of Electronic Thin Films and Integrated Devices, School of Optoelectronic Science and Engineering, University of Electronic Science and Technology of China (UESTC), Chengdu 610054, China

**Keywords:** perovskite solar cells, PEDOT:PSS, MoO_3_, hole transport layer, stability

## Abstract

Over the last ten years, there has been a remarkable enhancement in the power conversion efficiency (PCE) of perovskite solar cells (PSCs), with poly (3,4-ethylenedioxythiohene):poly (styrenesulfonate) (PEDOT:PSS) emerging as a prevalent choice for the hole transport layer (HTL). Nevertheless, the evolution of the widely utilized PEDOT:PSS HTL has not kept pace with the swift advancements in PSC technology, attributed to its suboptimal electrical conductivity, acidic nature, and inadequate electron-blocking performance. This study presents a novel approach to enhance the HTL by introducing molybdenum oxide (MoO_3_) into the PEDOT:PSS, leveraging the conductivity and solution processing compatibility of MoO_3_. Two methods for MoO_3_ integration were explored: an ammonium molybdate tetrahydrate (AMT) precursor and the direct addition of MoO_3_ nanoparticles. The carrier dynamics of PSCs modified by MoO_3_ are significantly optimized. Therefore, the PCE of the device modified by AMT and molybdenum oxide is increased to 18.23 and 19.64%, respectively, and the stability of the device is also improved. This study emphasizes the potential of MoO_3_ in contributing to the development of more efficient and stable PSCs.

## 1. Introduction

In the quest for sustainable energy sources, perovskite solar cells (PSCs) have emerged as a revolutionary technology, offering a promising solution with their exceptional power conversion efficiencies (PCEs), tunable bandgaps, and facile fabrication processes [1,2,3,4]. The rapid evolution of PSCs has seen their efficiencies soar to over 26% [5,6], rivaling those of traditional silicon-based photovoltaics. Central to this success is the strategic manipulation of the device architecture and material composition, particularly at the critical anode interface, where the hole transport layer (HTL) plays a pivotal role in determining the overall performance of the cell [7,8,9,10,11,12].

Poly (3,4-ethylenedioxythiophene):poly (styrenesulfonate) (PEDOT:PSS) has been widely employed as the HTL due to its favorable properties such as high work function, good film-forming ability, and compatibility with a range of processing techniques [13,14,15,16]. However, the intrinsic limitations of PEDOT:PSS include its acidic nature, relatively low conductivity, and susceptibility to moisture [17,18,19,20]. This has prompted researchers to explore alternative materials and strategies to enhance the HTL’s properties and, by extension, the efficiency and stability of PSCs.

Transition metal oxides, particularly molybdenum oxide (MoO_3_), have shown great potential as a component in HTLs due to their excellent conductivity and compatibility with solution processing [21,22]. MoO_3_ can be modified into the PEDOT:PSS to form a hybrid HTL that leverages the beneficial properties of both components [23]. The integration of MoO_3_ into PEDOT:PSS can be achieved through various methods, such as in situ thermal decomposition of a precursor like ammonium molybdate tetrahydrate (NH_4_)_6_Mo_7_O_24_·4H_2_O, or by direct addition of MoO_3_ nanoparticles (NPs) [24,25,26,27]. Both methods aim to enhance the HTL’s performance by increasing its conductivity, reducing its acidity, and improving its compatibility with the perovskite layer.

The thermal annealing approach using ammonium molybdate tetrahydrate (AMT) allows for the controlled formation of MoO_3_, which can lead to a more uniform and stable HTL [28]. This method offers precise control over the crystallinity and morphology of the resulting MoO_3_, which are critical factors in determining the HTL’s efficiency in extracting holes and blocking electrons. On the other hand, the direct addition of MoO_3_ NPs to the PEDOT:PSS matrix provides a more straightforward and potentially scalable method for HTL modification. This approach can result in an HTL with enhanced conductivity and a reduced tendency for moisture absorption, which are key factors in improving the long-term stability of PSCs.

In this work, we delved into the effects of these modifications on the photovoltaic performance of PSCs, with a particular focus on the PCE, short-circuit current density (J_SC_), and fill factor (FF). We also investigate the long-term stability of the devices under various environmental conditions, providing valuable insights into the practical viability of these modified HTLs. Through a series of experiments, we demonstrate that the incorporation of MoO_3_ into PEDOT:PSS can significantly enhance the performance of PSCs, achieving PCE that surpass those of devices with untreated PEDOT:PSS. This is mainly due to the modification of PEDOT:PSS film properties by introduction with MoO_3_, as well as the optimization of perovskite crystallinity. The results highlight the potential of these hybrid HTLs in promoting the commercialization of PSCs and contribute to the ongoing efforts to develop more efficient and stable solar energy technologies.

## 2. Results and Discussion

**Figure 1:**
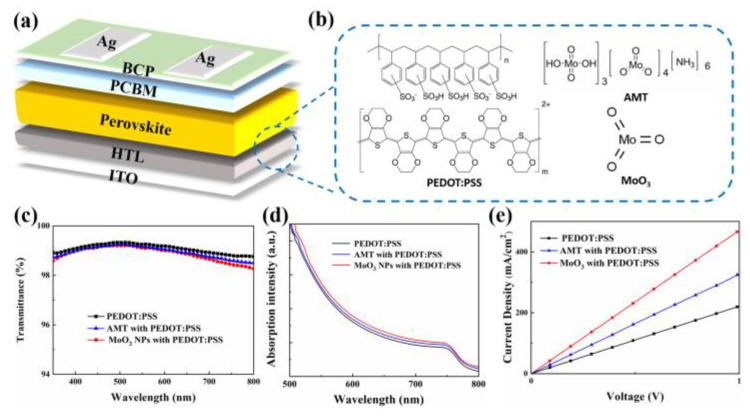
(**a**) Device structure and (**b**) chemical structure of materials for PSCs in this work; (**c**) transmission spectra of HTLs; (**d**) absorption spectra of perovskite on different HTLs; (**e**) conductivity test of different HTLs.

The device architecture and the molecular structures of HTL employed in this work are shown in Figure 1a,b. AMT has good solubility in water, while MoO_3_ nanoparticle ink also has excellent dispersion in water and isopropanol (as shown in Figure S1), so both can be fully mixed with PEDOT:PSS to form HTLs. To evaluate the impact of AMT or MoO_3_ NP incorporation on the characteristics of PEDOT:PSS films, we initially examined the UV-Vis transmission spectra of the PEDOT:PSS with different modifiers, as illustrated in Figure 1c. The findings indicate that the presence of AMT or MoO_3_ NPs will not significantly impact impact the transmittance of the PEDOT:PSS film, suggesting that a suitable concentration of AMT or MoO_3_ NPs does not impede photon absorption in perovskites constructed on PEDOT:PSS. This inference can also be confirmed by measuring the absorption spectra of perovskite active layers on different HTLs (Figure 1d).

Subsequently, we tested the effect of different introduction on the conductivity of PEDOT:PSS. The results are shown in Figure 1e. The device structure adopted is ITO/HTLs/Ag. From the figure, we can observe that after the addition of AMT and MoO_3_ NPs, the conductivity of PEDOT:PSS has been improved, and the increase in MoO_3_ NPs introduction is greater than that of AMT. The MoO_3_-NP- and AMT-modified PEDOT:PSS films have a conductivity of 1.39 × 10^−2^ S/cm and 9.89 × 10^−3^ S/cm, respectively, higher than that of pristine PEDOT:PSS film (6.51 × 10^−3^ S/cm). This shows that the introduction of MoO_3_ NPs and AMT can promote the carrier transport of HTLs. At the same time, we also tested the effect of different introduction on the surface morphology of PEDOT:PSS films by AFM. It can be seen from Figure S2 that the addition of AMT and MoO_3_ NPs slightly increases the surface roughness of PEDOT:PSS film, but the overall effect is not obvious, indicating that an appropriate amount of introduction will not damage the morphology of PEDOT:PSS film.

The overall device performance is significantly influenced by the interface quality between the PEDOT:PSS HTL and the perovskite active layer. To ascertain the AMT or MoO_3_ NP distribution across the PEDOT:PSS HTL surface, XPS analysis was conducted on the composite films and shown in Figure S3. After AMT or MoO_3_ NP introduction, the intensity of the Mo3d peak is significantly enhanced. This means that molybdenum trioxide is uniformly distributed on the surface of PEDOT:PSS, which may improve the contact interface between HTL and perovskite [29,30]. Furthermore, the water contact angle was measured to assess the impact of modifier on the PEDOT:PSS HTL surface wettability. As observed in Figure S4, after the introduction of AMT or MoO_3_ NPs, the water contact angle of PEDOT:PSS film does not change significantly (18°, 16°and 15°), which means that the introduction will make HTLs still maintain good hydrophilicity, which is conducive to the more uniform film formation of perovskite.

**Figure 2:**
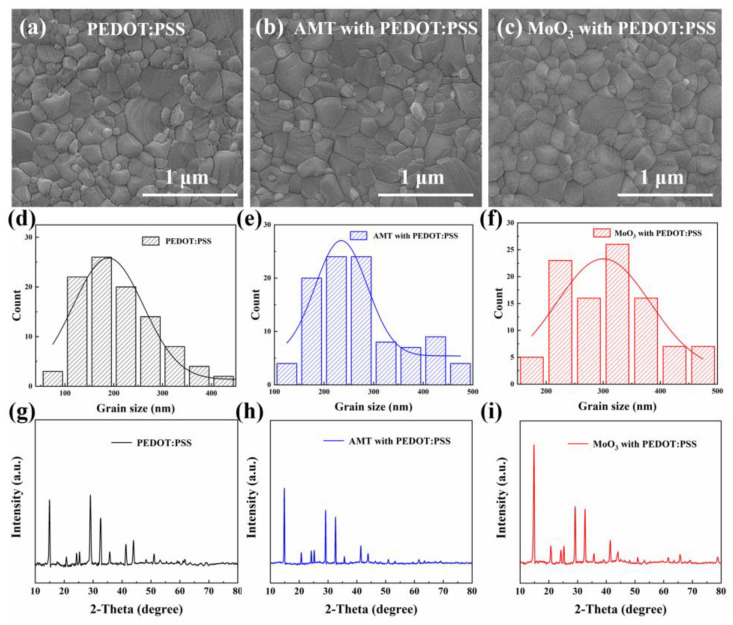
SEM images of perovskite films upon (**a**) pure PEDOT:PSS HTL, (**b**) AMT-modified PEDOT:PSS HTL and (**c**) MoO_3_-NP-modified PEDOT:PSS HTL; the histogram statistics of grain size for perovskite films with (**d**) pure PEDOT:PSS, (**e**) AMT-modified PEDOT:PSS and (**f**) MoO_3_-NP-modified PEDOT:PSS; XRD spectra of perovskite layer upon pure (**g**) PEDOT:PSS, (**h**) AMT-modified PEDOT:PSS and (**i**) MoO_3_-NP-modified PEDOT:PSS.

Next, in order to study the growth and crystallization of perovskite on different modified PEDOT:PSS HTLs, we fabricated perovskite films on different HTLs and tested thire SEM images. As shown in Figure 2a–c, the perovskite film prepared on pure PEDOT:PSS shows disordered grain size, many small grains are distributed on the film, and the grain boundary is also not clear, which is unfavorable to the performance of the PSCs. However, after AMT introduction, the perovskite grains become more ordered and the grain boundaries are clearer than those of pure PEDOT:PSS, but there are still a few small disordered grains upon the film. This phenomenon is significantly improved after the introduction of MoO_3_ NPs. The grain distribution and grain boundary of perovskite are better than the other two. After statistics of the average grain size data of perovskite (Figure 2d–f), the average grain size of pure PEDOT:PSS-based perovskite is 210 nm, while for AMT- and MoO_3_-NP-modified samples, the data are 270 and 310 nm respectively. Reducing the grain boundaries and increasing grain size can enhance the transport properties of the perovskite layer by minimizing charge carrier recombination, so as to improve the carrier transport efficiency of the calcium titanite layer [31,32]. In order to further study the crystallinity of perovskite films on different HTLs, we measured the XRD spectra of perovskite active layers on different HTLs. As shown in Figure 2g–i, the coordinate scales in the figures are unified. When PEDOT:PSS modified with MoO_3_ NPs, the intensity of diffraction peak became stronger than that of perovskite film on pure and AMT-modified PEDOT:PSS. It means that the addition of MoO_3_ NPs is conducive to the growth of perovskite grains upon HTLs, which is consistent with the above SEM results. This shows that the introduction of MoO_3_ NPs significantly improves the quality of the interface between HTL and perovskite, thus improving the growth and crystallization of perovskite.

**Figure 3:**
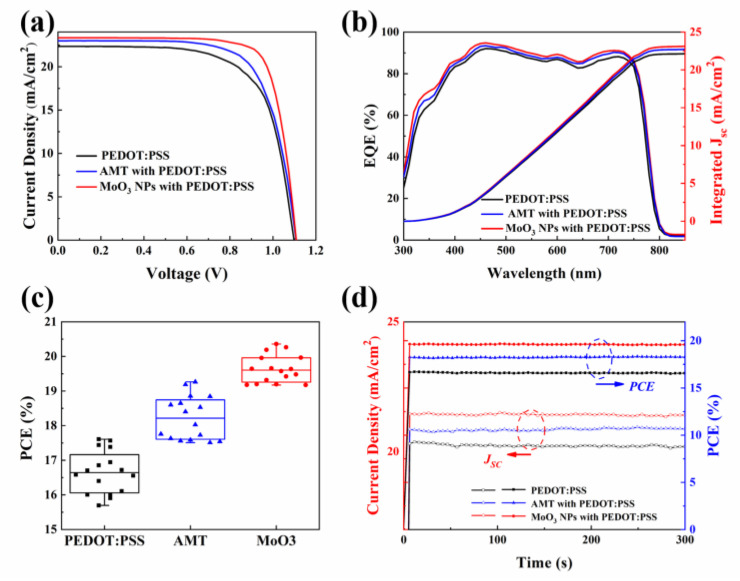
Control devices and modified devices. (**a**) Optimal *J–V* curves; (**b**) external quantum efficiency (EQE) spectra and integrated current density; (**c**) statistical analysis of the PCE of PSCs based on different HTLs (16 devices for each type); (**d**) steady-state output power and current density at maximum power point.

After analyzing the influence of AMT and MoO_3_ NPs on PEDOT:PSS, in order to verify the influence of different HTLs on device performance, we prepared PSCs based on different HTLs, and the structure of the devices is shown in Figure 1a. Figure 3a exhibits the current density–voltage (J–V) curves of PSCs, the corresponding operating parameters involving the open-circuit voltage (V_OC_), current density (J_SC_), fill factor (FF), and PCE are summarized in Table S1, and the performance data for different concentrations of modifiers are summarized in Tables S2 and S3. For devices using pure PEDOT:PSS as HTL, the J_SC_, V_OC_, and FF are 22.34 mA/cm^2^, 1.09 V, and 68.42%, respectively, and the corresponding PCE is 16.65%. In contrast, for devices based on AMT-modified HTL, the J_SC_, V_OC_, and FF are 22.98 mA/cm^2^, 1.10 V, and 72.13%, respectively, and the PCE is 18.23%. For devices using MoO_3_ NPs introduction, the J_SC_, V_OC_, and FF are 23.37 mA/cm^2^, 1.10 V, and 76.52%, respectively, and the corresponding PCE is 19.64%. It can be found that, after PEDOT:PSS introduction, the J_SC_ and FF of PSCs have been improved, while being modified with MoO_3_ NPs, and the device performance has been greatly improved. The improved conductivity of modified PEDOT:PSS and the minimized charge recombination due to the enhanced grain growth quality of the perovskite film are responsible for the increased J_SC_ and FF. The V_OC_ value remains almost unchanged. Furthermore, the hysteresis behavior of PSCs has been examined. As depicted in Table S1, devices with pristine PEDOT:PSS such as the HTL exhibit minimal hysteresis. For modified devices, there is an insignificant variation between reverse and forward scans, which is comparable to unmodified ones. The hysteresis indices of PSCs are 10.4% (PEDOT:PSS), 5.9% (AMT modified), and 4.8% (NPs modified), respectively. This reduction in hysteresis is likely due to the enhanced charge transport and the reduced charge recombination rate in modified PSCs, resulting in a more symmetrical electron and hole mobility [33].

It should be highlighted that the EQE results and integrated J_SC_ derived from the EQE curve aligns with that from the J–V curves, as depicted in Figure 3b (the integrated J_SC_ for control, AMT- and MoO_3_-NP-modified devices are 22.13, 22.68 and 23.10 mA/cm^2^, respectively). The PCE distribution data in Figure 3c indicates that the device with MoO_3_ NP modification exhibits enhanced repeatability and statistical reliability in comparison to the oure PEDOT:PSS and AMT-modified counterparts. This may be due to the better crystallinity of perovskite modified by MoO_3_ NPs. Furthermore, to assess the output stability of PSCs, we determined the constant photocurrent at the maximum power point of the devices (illustrated in Figure 3d). It could be noticed that the devices, both with and without introduction, sustain consistent J_SC_ and PCE under their maximum output power voltage. These outcomes align with the data derived from the J–V curve examination, thereby confirming the devices’ output reliability based on different HTLs.

**Figure 4:**
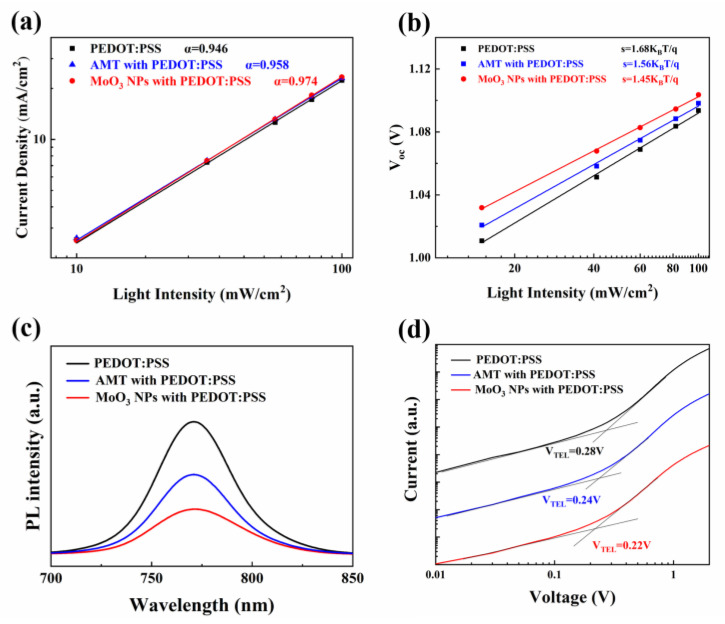
(**a**) Light intensity dependence of *J_SC_* of PSCs with different HTLs; (**b**) *V_OC_* change of the device under different light intensity; (**c**) steady-state PL of perovskite films upon different HTLs; (**d**) dark current–voltage characteristics of hole-only devices.

The performance of PSCs is significantly influenced by bimolecular recombination within the devices. Figure 4a illustrates the correlation between *J_SC_* and the intensity of light, *P_light_*. This interrelation can be articulated through Equation (1).

(1) $$J_{SC}\propto {P_{light}}^{\alpha }$$

The *α* indicator is associated with the extent of compositeness. In particular, as the *α* approaches 1, the rate of bimolecular recombination diminishes. Figure 4a illustrates that the *α* for pure, AMT-modified and MoO_3_-NP-modified PEDOT:PSS are 0.946, 0.958 and 0.974, respectively. This finding indicates that the incorporation of two kinds of modifiers leads to a more pronounced reduction in bimolecular recombination within the perovskite layer compared to the unmodified counterparts.

To investigate how AMT and MoO_3_ NPs specifically affect the trap-assisted recombination in PSCs, we established a correlation between the V_OC_ and the ln (Plight) slope, depicted in Figure 3b. Ideally, a reduced trap-assisted recombination would result in a gradient approaching 1 K_B_T/q (with K_B_ being the Boltzmann constant, T signifying the absolute temperature, and q denoting the elementary charge). The findings indicate that the PEDOT:PSS-based device has a slope of 1.68 K_B_T/q, whereas devices incorporating an AMT or MoO_3_ NP composite HTL exhibit decreased slopes of 1.56 and 1.45 K_B_T/q. This suggests that incorporating AMT or MoO_3_ NPs can significantly suppress trap-assisted recombination, potentially serving as a critical element in enhancing the device’s FF [33].

The interaction of charge carriers at the interface of the perovskite layer and the HTL significantly influences the efficiency of PSCs. To assess the effectiveness of charge extraction and the efficiency of charge transfer for various HTLs, steady-state photoluminescence (PL) measurements were conducted on perovskite layers that were coated with both unmodified and modified PEDOT:PSS films. As depicted in Figure 4c, the PL intensity for samples with unmodified PEDOT:PSS is higher than that for those with AMT- and MoO_3_-NP-modified samples, suggesting a superior quenching effect compared to the non-modified ones. This enhanced quenching is likely due to a reduced rate of charge recombination, which aids in the extraction of charge carriers from the perovskite layer to the HTL and promotes more efficient charge transfer to the cathode [34].

In order to delve deeper into the impact of various HTLs on the defect state density within the perovskite layer, one can determine the trap state densities of these films employing the space charge limited current (SCLC) technique [35]. The configuration of the device is composed of ITO/HTL/perovskite/Spiro/Ag. The formula for calculating the defect state density, denoted as *N_trap_*, for the perovskite film is outlined in Equation (2).

(2) $$V_{TFL}=\frac{qN_{trap}L^{2}}{2\varepsilon \varepsilon_{0}}$$

The defect filling limit voltage is denoted as *V_TFL_*, with *L* representing the perovskite film’s thickness, *ε* signifying its relative dielectric constant, and *ε_0_* referring to the vacuum’s dielectric constant. The single-hole device’s effective area measures 4.5 mm^2^. Figure 4d illustrates that the *V_TFL_* for PSCs with pure, AMT-modified and MoO_3_-NP-modified PEDOT:PSS are 0.28, 0.24 and 0.22 V, respectively. Calculations reveal that the trap density (*N_trap_*) for the standard device using pristine PEDOT:PSS is 9.72 × 10^15^ cm^−3^. In contrast, the *N_trap_* for the hybrid HTL modified with AMT is decreased to 8.33 × 10^15^ cm^−3^, this value is markedly decreased to 7.64 × 10^15^ cm^−3^ for MoO_3_ NPs modified devices. This reduction is likely due to the higher purity, reduced defect occurrence, and lower defect state density in perovskite films developed on the MoO_3_ NPs composite HTL. A lower defect state density is advantageous for suppressing charge recombination within the perovskite, thereby enhancing device performance [36,37].

**Figure 5:**
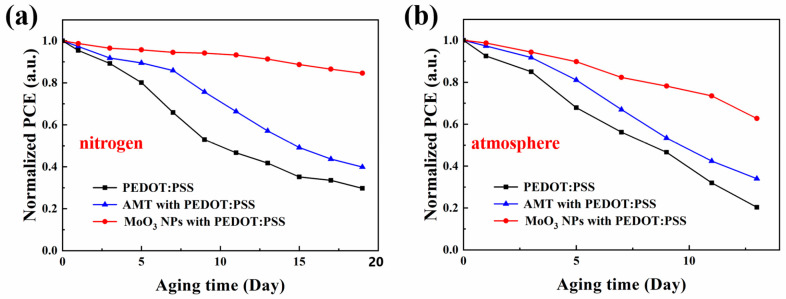
Stability test of PSCs based on different HTLs in (**a**) nitrogen and (**b**) air atmosphere.

In order to assess the impact of the incorporated AMT and MoO_3_ NPs on the stability of PSCs, we conducted tests on the stability of PSCs with various HTLs in different storage conditions. These tests were carried out in an unsealed state at room temperature, under conditions of nitrogen atmosphere and ambient humidity levels (ranging from 40% to 80%), as depicted in Figure 5a,b. For devices stored in nitrogen atmosphere, by using MoO_3_ NPs as modifier in PEDOT:PSS, after a 19-day storage period, the devices exhibited improved stability, notably surpassing those that employed pure PEDOT:PSS-based HTL. This may be because the introduction of molybdenum trioxide reduces the acidity of PEDOT:PSS. At the same time, we also noticed that the introduction of AMT did not significantly improve the stability of the device in nitrogen atmosphere.

For devices stored in the atmosphere, as illustrated in Figure 5b, devices with an HTL containing MoO_3_ NPs maintained 63% of their initial PCE after 13-day exposure, whereas devices with pure PEDOT:PSS HTL only retained 20% of their initial efficiency. Furthermore, devices with AMT in the HTL showed 34% of their original efficiency. The above discussion shows that using MoO_3_-NP-modified PEDOT:PSS as HTL can not only improve the lifetime of PSCs in nitrogen, but also optimize the stability of devices in atmospheric environment.

## 3. Materials and Methods

### 3.1. Materials and Solution Preparation

The MoO_3_ nanoparticle ink was obtained from Sigma Aldrich, Michigan, MI, USA (2.3–2.7 wt.%, crystalline MoO_3_ in ethanol). The AMT powder was obtained from Sigma Aldrich (≤0.005% insolubles). All other perovskite and transport layers (including PEDOT:PSS) were purchased from P-oled, Xian, China. To prepare the AMT- and MoO_3_-NP-modified PEDOT:PSS solution, we dissolved AMT into DI water to get a solution (20 mg/mL) and dissolved the MoO_3_ NPs dispersion (volume percent from 4 to 16 vol % ) into PEDOT:PSS to achieve a composite solution. Then, the AMT and MoO_3_ NPs solution can be added to PEDOT:PSS to prepare mixed solution.

FA_0.95_MA_0.05_PbI_2.85_Br_0.15_ perovskite precursor was prepared by dissolving FAI (0.2451 g), PbI2 (0.6569 g), MABr (0.0084 g), PbBr2 (0.0275 g), and MACl (0.0330 g) in DMSO (0.111 mL) and DMF (0.889 mL). This mixture was agitated continuously throughout the night to yield the final perovskite precursor solution. PC_61_BM (Polymer Light Technology Corp. Xian, China) solution in o-dichlorobenzene (DCB) with 22 mg/mL. Bathocuproine (BCP) was dissolved in methanol with 2 mg/mL to obtain a solution.

### 3.2. PSCs Fabrication

The substrates underwent a sequential cleaning process in an ultrasonic cleaner, using detergent, acetone, deionized water, and isopropyl alcohol for 15 min per solution, followed by drying at a temperature of 80 °C for 60 min before application. Subsequently, the cleaned substrates were treated with UV-ozone for 10 min. Mixed and unmixed PEDOT:PSS solution was spin-coated on the top of ITO-coated glass substrates and baked at 150 °C for 1 h in ambient.

For the fabrication of the perovskite film, a precursor mixture was spun at 4000 rpm for 20 s. During this spin-coating, 200 μL of chlorobenzene was dropped onto the rotating substrate 6 s prior to completion. Following this, the perovskite films were thermally treated at 100 °C for 10 min within a glove box. After reaching ambient temperature, a PC_61_BM layer was applied via spin-coating at 1600 rpm for 30 s. Once the solvent had volatilized, a BCP layer was deposited upon PC_61_BM layer via spin-coating at 3000 rpm for 30 s. Subsequently, the devices were transferred to a vacuum chamber for the deposition of an 100 nm thick Ag layer as the cathode, conducted under a pressure less than 1 × 10^−3^ Pa.

### 3.3. Characterization and Measurement

PSCs underwent testing in standard environmental conditions at ambient temperature. The J–V characteristics were recorded using an artificial light source (CHF-XM35, Beijing Trusttech, Beijing, China) at 100 mW/cm^2^. The J–V curves were obtained from −0.5 V to 1.5 V with a 0.01 V step. The tested PSCs covered an area of 4.5 mm^2^. EQE measurements were conducted using a monochromator-equipped Solar Cell Scan 100 from Zolix instruments Co., Ltd., Beijing, China. Active layer absorption was assessed with a Shimadzu UV1700 system on quartz substrates.

AFM analysis (Agilent 5500, Santa Clara, CA, USA) was performed to examine surface morphology. Surface SEM images were captured using an SEM (FEI Inspect F50, Thermo Scientific, Waltham, MA, USA). The crystallinity of perovskite was analyzed using XRD (Bruker AXS D2 Phaser, Billerica, MA, USA), employing a monochromatic Cu Kα (λ = 0.154 nm) excitation source with scanning voltage = 40 kV and scanning current = 40 mA. PL and time-resolved PL data were acquired with a Jobin Yvon Fluorolog-3-TAU spectrometer excited at 500 nm. XPS analysis was conducted with a Thermo Scientific K-Alpha + system, Waltham, MA, USA use of an Al Kα X-ray source.

## 4. Conclusions

In conclusion, we had developed a potent approach to enhance the inherent characteristics of PEDOT:PSS through the integration of transition metal oxides, MoO_3_, specifically by two introduction methods. This method leads to higher electrical conductivity, reduced carrier recombination, and a more effective barrier against electron leakage in the PEDOT:PSS film. The introduction of MoO_3_ improves the crystallinity of perovskite on PEDOT:PSS surface, forms a better active layer, and also regulates the interface contact between HTL and perovskite so that the carrier dynamics are optimized, which in turn enhances the J_SC_ and FF. Utilizing the MoO_3_-NPs-PEDOT:PSS composite, we achieved an outstanding average PCE of 19.64% for PSCs and 18.23% for those with AMT-PEDOT:PSS composite. The modified PSCs show excellent reproducibility and stability, marking a significant improvement compared with the control devices. These results significantly deepen our comprehension of HTL design, which is crucial for the development of high-performance PSCs.

## Data Availability

The original contributions presented in the study are included in the article/Supplementary Materials, and further inquiries can be directed to the corresponding author.

## Supplementary Materials

The following supporting information can be downloaded at: https://www.mdpi.com/article/10.3390/molecules29215064/s1, Figure S1: Images of MoO3 NPs ink dispersion in water and IPA; Figure S2: AFM images of (a) pure PEDOT:PSS, (b) AMT doped PEDOT:PSS and (c) MoO_3_ NPs doped PEDOT:PSS; Figure S3: Mo3d peak of different HTLs; Figure S4: Water contact angle of (a) pure PEDOT:PSS (b) AMT doped PEDOT:PSS and (c) MoO_3_ NPs doped PEDOT:PSS; Figure S5: PH value of (a) pure PEDOT:PSS (b) AMT doped PEDOT:PSS and (c) MoO_3_ NPs doped PEDOT:PSS; Table S1: Key photovoltaic parameters of PSCs with different ETLs; Table S2: Key photovoltaic parameters of PSCs with different AMT ratios; Table S3: Key photovoltaic parameters of PSCs with different MoO_3_ NPs ratios.

## References

[1] Kim J.Y., Lee J.-W., Jung H.S., Shin H., Park N.-G. (2020). High-Efficiency Perovskite Solar Cells. Chem. Rev..

[2] Liu D., Kelly T.L. (2014). Perovskite solar cells with a planar heterojunction structure prepared using room-temperature solution processing techniques. Nat. Photonics..

[3] Jeon N.J., Noh J.H., Kim Y.C., Yang W.S., Ryu S., Seok S.I. (2014). Solvent engineering for high-performance inorganic-organic hybrid perovskite solar cells. Nat. Mater..

[4] Liu M., Johnston M.B., Snaith H.J. (2013). Efficient planar heterojunction perovskite solar cells by vapour deposition. Nature.

[5] Zhou J., Tan L., Liu Y., Li H., Liu X., Li M., Wang S., Zhang Y., Jiang C., Hua R. (2024). Highly efficient and stable perovskite solar cells via a multifunctional hole transporting material. Joule.

[6] Liu S., Li J., Xiao W., Chen R., Sun Z., Zhang Y., Lei X., Hu S., Kober-Czerny M., Wang J. (2024). Buried interface molecular hybrid for inverted perovskite solar cells. Nature.

[7] Arora N., Dar M.I., Hinderhofer A., Pellet N., Schreiber F., Zakeeruddin S.M., Graetzel M. (2017). Perovskite solar cells with CuSCN hole extraction layers yield stabilized efficiencies greater than 20%. Science.

[8] Habisreutinger S.N., Leijtens T., Eperon G.E., Stranks S.D., Nicholas R.J., Snaith H.J. (2014). Carbon Nanotube/Polymer Composites as a Highly Stable Hole Collection Layer in Perovskite Solar Cells. Nano. Lett..

[9] Jeon N.J., Na H., Jung E.H., Yang T.-Y., Lee Y.G., Kim G., Shin H.-W., Seok S.I., Lee J., Seo J. (2018). A fluorene-terminated hole-transporting material for highly efficient and stable perovskite solar cells. Nat. Energy.

[10] Jung E.H., Jeon N.J., Park E.Y., Moon C.S., Shin T.J., Yang T.-Y., Noh J.H., Seo J. (2019). Efficient, stable and scalable perovskite solar cells using poly(3-hexylthiophene). Nature.

[11] Chen X., Cheng J., He L., Zhao L., Zhang C., Pang A., Li J. (2023). Hole Transport Materials for Tin-Based Perovskite Solar Cells: Properties, Progress, Prospects. Molecules.

[12] Vailassery J., Sun S.-S. (2023). Recent Progress of Helicene Type Hole-Transporting Materials for Perovskite Solar Cells. Molecules.

[13] Chen W.-Y., Deng L.-L., Dai S.-M., Wang X., Tian C.-B., Zhan X.-X., Xie S.-Y., Huang R.-B., Zheng L.-S. (2015). Low-cost solution-processed copper iodide as an alternative to PEDOT:PSS hole transport layer for efficient and stable inverted planar heterojunction perovskite solar cells. J. Mater. Chem. A.

[14] Han W., Ren G., Liu J., Li Z., Bao H., Liu C., Guo W. (2020). Recent Progress of Inverted Perovskite Solar Cells with a Modified PEDOT:PSS Hole Transport Layer. ACS Appl. Mater. Int..

[15] Liu D., Li Y., Yuan J., Hong Q., Shi G., Yuan D., Wei J., Huang C., Tang J., Fung M.-K. (2017). Improved performance of inverted planar perovskite solar cells with F4-TCNQ modified PEDOT:PSS hole transport layers. J. Mater. Chem. A.

[16] Zuo C., Ding L. (2017). Modified PEDOT Layer Makes a 1.52 V Voc for Perovskite/PCBM Solar Cells. Adv. Energy Mater..

[17] Huang D., Goh T., Kong J., Zheng Y., Zhao S., Xu Z., Taylor A.D. (2017). Perovskite solar cells with a DMSO-treated PEDOT: PSS hole transport layer exhibit higher photovoltaic performance and enhanced durability. Nanoscale.

[18] Huang X., Wang K., Yi C., Meng T., Gong X. (2016). Efficient perovskite hybrid solar cells by highly electrical conductive PEDOT:PSS hole transport layer. Adv. Energy Mater..

[19] Qi Y., Almtiri M., Giri H., Jha S., Ma G., Shaik A.K., Zhang Q., Pradhan N., Gu X., Hammer N.I. (2022). Evaluation of the passivation effects of PEDOT:PSS on inverted perovskite solar cells. Adv. Energy Mater..

[20] Yang Y., Yao Y., Li Y., Zhao X., Cheng W., Chen B., Chen L., Li P., Tang S. (2023). Application of arginine-modified PEDOT:PSS as a hole transfer layer in perovskite solar cells. J. Mater. Chem. C.

[21] Hou F., Su Z., Jin F., Yan X., Wang L., Zhao H., Zhu J., Chu B., Li W. (2015). Efficient and stable planar heterojunction perovskite solar cells with an MoO_3_/PEDOT:PSS hole transporting layer. Nanoscale.

[22] Liu F., Shao S., Guo X., Zhao Y., Xie Z. (2010). Efficient polymer photovoltaic cells using solution-processed MoO_3_ as anode buffer layer. Sol. Energy Mater. Sol. Cells.

[23] Kim D.B., Yu J.C., Nam Y.S., Kim D.W., Jung E.D., Lee S.Y., Lee S., Park J.H., Lee A.-Y., Lee B.R. (2016). Improved performance of perovskite light-emitting diodes using a PEDOT:PSS and MoO_3_ composite layer. J. Mater. Chem. C.

[24] Liu Z., Hui S., Wang N. (2017). Improved performance and stability of inverted polymer solar cells with ammonium heptamolybdate acted as hole extraction layers via thermal annealing method. J. Lumin..

[25] Murase S., Yang Y. (2012). Solution processed MoO_3_ interfacial layer for organic photovoltaics prepared by a facile synthesis method. Adv. Mater..

[26] Zhao B., Chung S., Zhang M., Wei W., Zhu C., Deng C., Cho K., Kan Z. (2023). 18.9% efficiency binary organic solar cells enabled by regulating the intrinsic properties of PEDOT:PSS. Adv. Funct. Mater..

[27] Wang Y., Luo Q., Wu N., Wang Q., Zhu H., Chen L., Li Y.-Q., Luo L., Ma C.-Q. (2015). Solution-processed MoO_3_:PEDOT:PSS hybrid hole transporting layer for inverted polymer solar cells. ACS Appl. Mater. Int..

[28] Niu S., Xing S., Zhan X., Liu Z., Wang N., Zheng W. (2018). Low-temperature solution-processed molybdenum oxide thin film as ITO modified layer for polymer solar cells. Sol. Energy.

[29] Xie F., Choy W.C.H., Wang C., Li X., Zhang S., Hou J. (2013). Low-temperature solution-processed hydrogen molybdenum and vanadium bronzes for an efficient hole-transport layer in organic electronics. Adv. Mater..

[30] Wang C., Su Z., Chen L., Zhang H., Hui W., Liang D., Zheng G., Zhang L., Tang Z., Wen W. (2022). MoO_3_ modified PTAA for high-performance inverted perovskite solar cells. Appl. Surf. Sci..

[31] Tingare Y.S., Hsu Y.-C., Lin J.-D., Su C., Wang W.-C., Wang S.-H., Lai S.-Y., Wu Z.-T., Lin J.-H., Wang H.-H. (2024). Zinc complex-based hole transporting material for perovskite solar cell applications. J. Mater. Chem. C.

[32] Lin Y., Zhang X., Lu J., Lin X., Lu Y., Li X., Tu S. (2024). Improvement in dibenzofuran-based hole transport materials for flexible perovskite solar cells. Molecules.

[33] Wang Z., Gao H., Wu D., Meng J., Deng J., Cui M. (2024). Defects and defect passivation in perovskite solar cells. Molecules.

[34] Zhou H., Chen Q., Li G., Luo S., Song T.-B., Duan H.-S., Hong Z., You J., Liu Y., Yang Y. (2014). Interface engineering of highly efficient perovskite solar cells. Science.

[35] Shen J., Ge X., Ge Q., Li N., Wang Y., Liu X., Tao J., He T., Yang S. (2024). Improvement of photovoltaic performance of perovskite solar cells by synergistic modulation of SnO2 and perovskite via interfacial modification. ACS Appl. Mater. Int..

[36] Zheng D., Peng R., Wang G., Logsdon J.L., Wang B., Hu X., Chen Y., Dravid V.P., Wasielewski M.R., Yu J. (2019). Simultaneous bottom-up interfacial and bulk defect passivation in highly efficient planar perovskite solar cells using nonconjugated small-molecule electrolytes. Adv. Mater..

[37] Zheng D., Wang G., Huang W., Wang B., Ke W., Logsdon J.L., Wang H., Wang Z., Zhu W., Yu J. (2019). Combustion synthesized zinc oxide electron-transport layers for efficient and stable perovskite solar cells. Adv. Funct. Mater..

