# Supplementary material for: Molybdenum-Oxide-Modified PEDOT:PSS as Efficient Hole Transport Layer in Perovskite Solar Cells
Source: Molecules. 2024 Oct 26;29(21):5064. doi: 10.3390/molecules29215064 (PMC11547414; doi:10.3390/molecules29215064)
Supplement: Supplementary file 1 [file molecules-29-05064-s001.zip › molecules-3268981-supplementary.pdf]

Supporting Information

Molybdenum-Oxide-Modified PEDOT:PSS as Efficient Hole Transport Layer in Perovskite Solar Cells

Pu Fan [1], Zhipeng Zhou [1,2], Jianghao Tian [1,2] and Junsheng Yu [1,2,*]

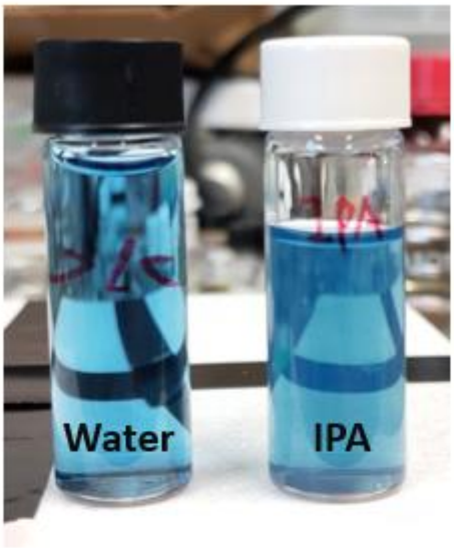

Figure S1. Images of MoO_3 NPs ink dispersion in water and IPA.

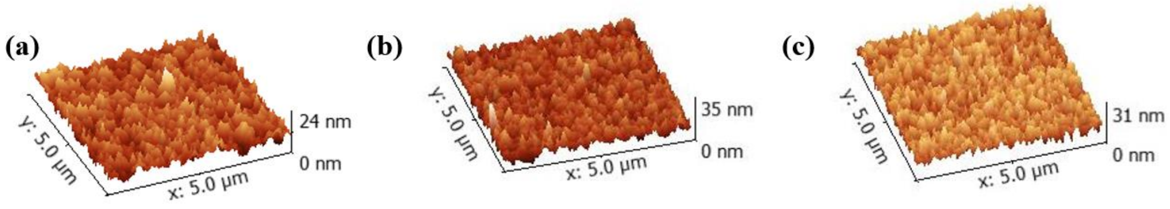

RMS= 2.42 nm RMS= 2.68 nm RMS= 2.94 nm

Figure S2. AFM images of (a) pure PEDOT:PSS, (b) AMT doped PEDOT:PSS and (c) MoO_3 NPs doped PEDOT:PSS.

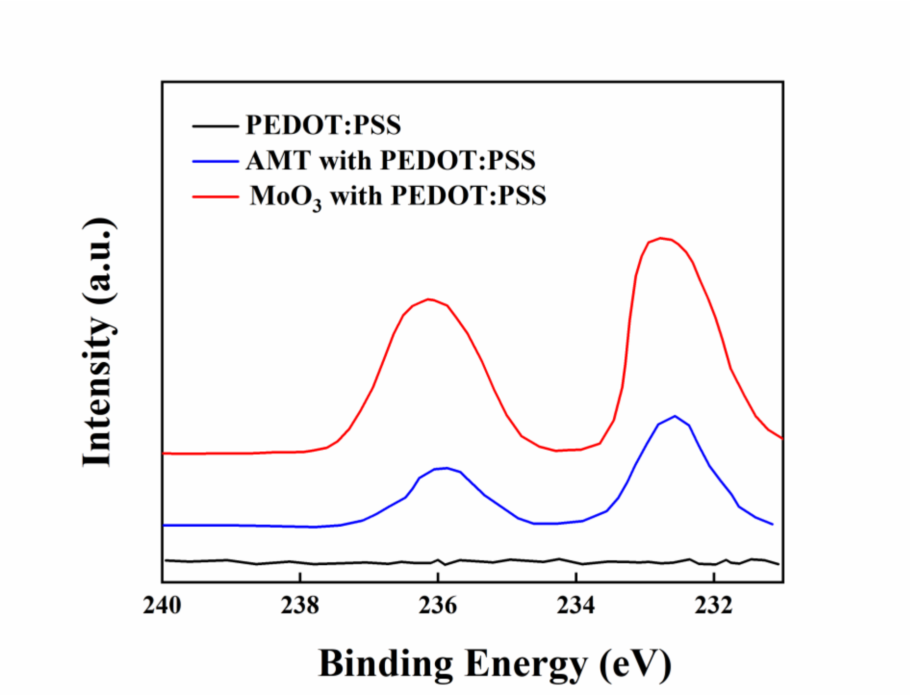

Figure S3. Mo3d peak of different HTLs.

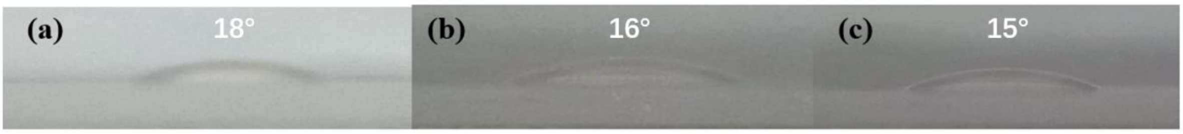

Figure S4. Water contact angle of (a) pure PEDOT:PSS (b) AMT doped PEDOT:PSS and (c) MoO_3 NPs doped PEDOT:PSS.

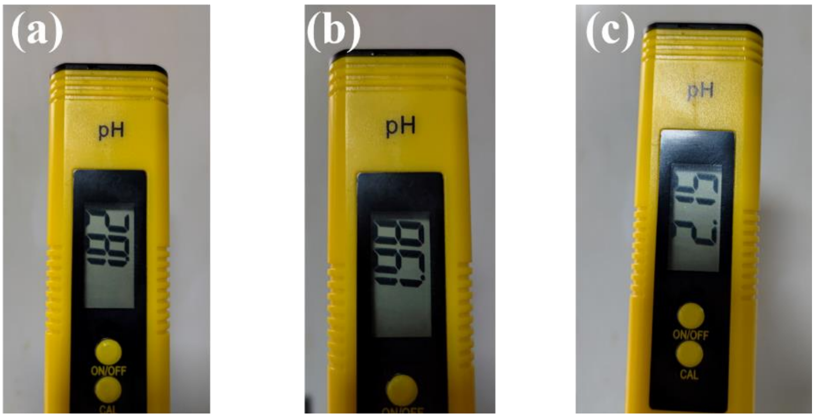

Figure S5. PH value of (a) pure PEDOT:PSS (b) AMT doped PEDOT:PSS and (c) MoO_3 NPs doped PEDOT:PSS.

Table S1. Key photovoltaic parameters of PSCs with different ETLs[a)]

ETL		V_{OC} (V)	J_{SC} (mA cm^{-2})	FF (%)	PCE (%)
PEDOT:PSS	RS	1.09	22.34	68.42	16.65
	FS	1.07	21.74	64.16	14.92
AMT with PEDOT:PSS	RS	1.10	22.98	72.13	18.23
	FS	1.09	22.53	69.83	17.15
MoO3 NPs with PEDOT:PSS	RS	1.10	23.37	76.52	19.64
	FS	1.09	23.06	74.39	18.70

[a)]All parameters are average value collected from 16 devices.

Table S2. Key photovoltaic parameters of PSCs with different AMT ratios[a]

AMT ratio (wt %)	V_{OC} (V)	J_{SC} (mA/cm^2)	FF (%)	PCE (%)
0 (control)	1.09	22.34	68.42	16.65
2	1.09	22.61	69.93	17.23
5	1.10	22.98	72.13	18.23
8	1.10	22.43	71.32	17.59
10	1.10	21.87	70.33	16.91

[a]All parameters are average value collected from 16 devices.

Table S3. Key photovoltaic parameters of PSCs with different MoO_3 NPs ratios[a]

MoO_3 NPs ratio (vol %)	V_{OC} (V)	J_{SC} (mA/cm^2)	FF (%)	PCE (%)
0 (control)	1.09	22.34	68.42	16.65
4	1.10	22.78	72.13	18.07
8	1.10	23.37	76.52	19.64
12	1.10	22.96	73.16	18.47
16	1.10	22.57	71.73	17.80

[a]All parameters are average value collected from 16 devices.
